# A Modified Pseudoisochromatic Ishihara Colour Vision Test Based on Eastern Arabic Numerals

**Published:** 2013

**Authors:** Fatemeh Heidary, Reza Gharebaghi

**Affiliations:** Shahid Beheshti University of Medical Sciences, Medical School, Tehran, Iran

**Keywords:** Colour blindness, Pseudoisochromatic Ishihara plates, Eastern Arabic numerals

## Abstract

Congenital colour vision defects affect about 8% and 0.5% of the male and female population, respectively. Pseudoisochromatic Ishihara plates have shown to be successful in an early diagnosis of colour vision defects. This commonly used colour vision test was initially intended to identify those who suffered from red-green aspect of congenital colour blindness; however, it may be of use to reveal acquired colour vision defects as well. Despite the Ishihara plates’ value, there are a number of shortcomings in their current layout. We proposing a new colour plate modified from original Ishihara test. To best assist illiterates who are not able to read English, standard Ishihara plates have been translated to Eastern Arabic numerals, which are used in most parts of the Middle East, Central Asia and Africa populations. The purpose of the present modification was to present the new plates to these regions, but more research and study is required to work on the validity, reliability, and repeatability of these new plates.

## INTRODUCTION

Visual impairment and blindness has remained as one of the most important health issues in the Eastern Mediterranean countries [1], as there are 40.5 million living with visual impairment and 5 million afflicted with blindness which in some cases could have been prevented with prophylactic and screening measures. World Health Organization has estimated the prevalence and causes of visual impairment in the region [2].

Congenital colour vision defects affect 8% and 0.5% of males and females, respectively on a global basis. The high prevalence of colour blindness necessitates early diagnosis, since these individuals with this disorder cannot accurately make colour discrimination which will impact their performance both personally and professionally [3]. Therefore, colour vision assessment is essential in a complete visual examination. Many tests have been developed and distributed worldwide to diagnose colour vision defects. Colour defect tests are performed mostly for three goals, the first for screening for the presence of congenital or acquired defects, the second to diagnose the type and severity of the defects and the third to assess the impact of the defect on a specific profession or employment [4]. Generally speaking, a precise, easy, and cost effective test is needed to diagnose visual colour defects accurately [5].

Many tests are used clinically to diagnose visual colour defects, these tests range from simple Ishihara plates to more complex grading tests including Farnsworth-Munsell 100-Hue test (FM 100-Hue), the D-15 Farnsworth-Munsell (D-15), and an anomaloscope for more accurate and precise diagnosis [6-7]. 

Pseudoisochromic plates are the most popular tests and are used widely for screening purposes [8]. However, the goal of this paper is not to evaluate the advantages and disadvantages of different diagnostic tests although each test has its own limitations.

The commonly used Ishihara test was first designed to evaluate and identify red-green colour vision defects. Of course, this test is not comprehensive in scope and sometimes some individuals with the colour vision defects may be missed [7,9]. The Ishihara test includes one demonstration plate and 14 screening plates, which present three numeral designs of vanishing, transformation and hidden digits. Transformation with cards number of 2 to 7, vanishing design 8 to 13 and hidden 14 and 15 compose the Ishihara test [10].

On the other hand, many Arabic scripts and words are used in other languages in Islamic world, like Persian, Turkish, Malay, Hausa, Somali, Urdu, Hindi, Bengali, Pashto, Kazakh, and Bosnian. Mostly the Middle East, Central Asia and some African people are able to read easily Arabic numerals rather than English scripts and words. In this manuscript we present a new colour vision test based on standard pseudoisochromatic Ishihara plates to be used in the aforementioned regions more effectively.

## HYPOTHESES/IDEA

Optometrists and ophthalmologists use specific tests in many different clinical circumstances. Ease of use, validity and reliability, and also speed are among the most important factors affecting the choice of which test to administer. Clinically, there has been a controversy regarding the Ishihara test with English characters in the Middle East countries especially for those who are illiterate and/or children. To overcome this problem, Arabic numerals optotype were substituted in the new plates. 

In other words, English format of pseudoisochromatic Ishihara plates were translated, modified and newly designed based on Eastern Arabic Numerals to assist illiterates and children who are not able to read English. 

In Middle East, most children are able to read Arabic numerals prior to school age since they have been educated either in kindergarten or by their families. In addition, illiterates are more able to recognize Arabic numerals rather than English characters in these regions. 

**Figure 1:**
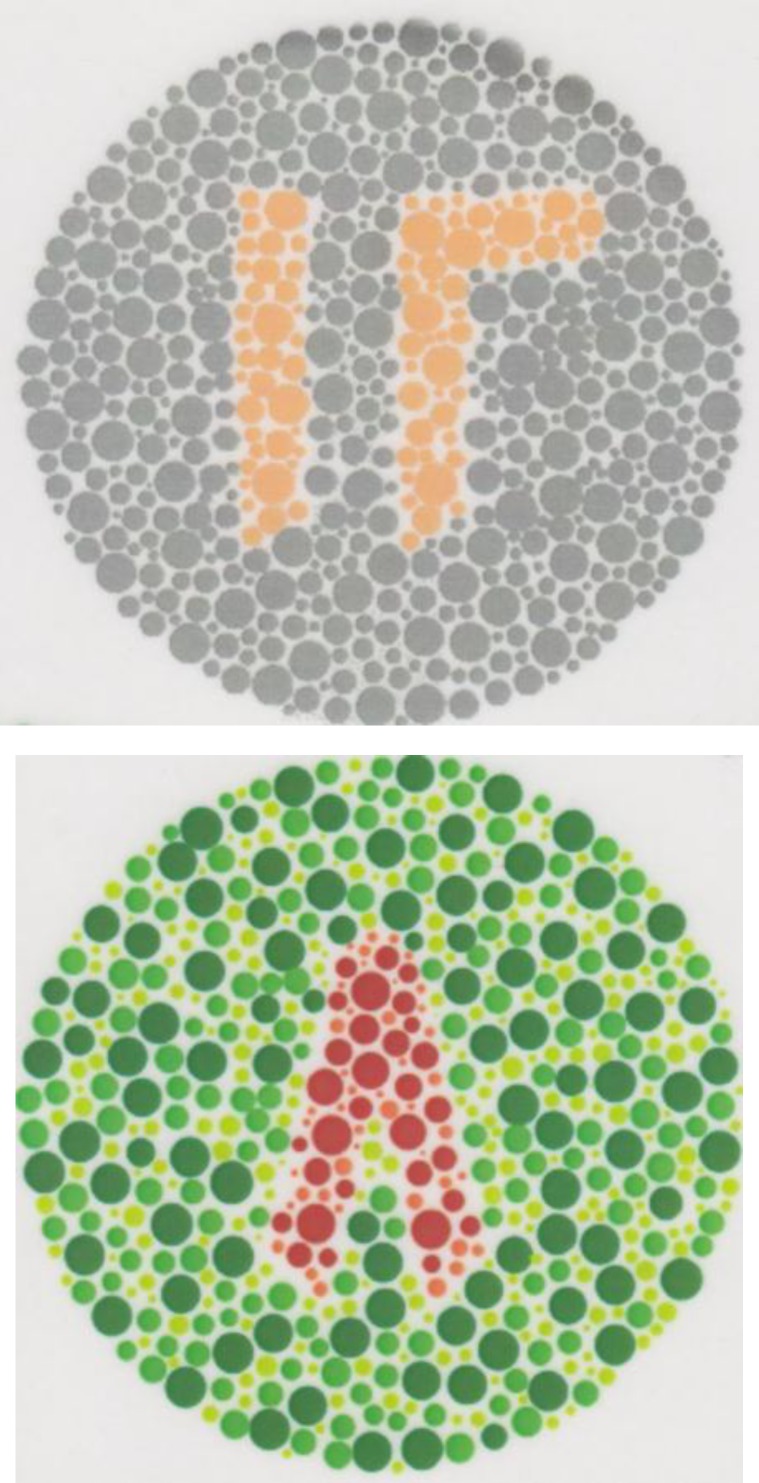
Demonstration colour vision plate based on Eastern Arabic numerals showing number 12 in the upper plate and number 8 in lower plate on random pattern of differently sized dots

Historically, numbers in each plate were surrounded by a random pattern of differently sized dots. In the new test, demonstration (figure 1), disappearing, alteration, and hidden diagnostic plates were used accordingly. The author recommended a standard Ishihara grade of illumination for measuring or screening purposes, and numbers were constructed on a common square grid. All Arabic numerals were evaluated to choose the best ones as well as the common optotypes between several languages in the region to be used for designing the colour vision plates. These designed plates are only used for adult illiterates and children who are not able to read English in the region.

## DISCUSSION

Our objective was to design a new colour vision test however more studies are needed to be performed in order to evaluate the validity and reliability of the plates in different geographical regions. 

There are a number of available colour vision tests but all have some limitations, and there is a need for a reliable test that can be performed without any specific clinical settings, expensive tools or a specially trained technician. This test must be quick to administer and easily scored to assess the type and severity of colour vision defects [7]. Trial subjects of the new test revealed promising results regarding convenience of use, portability, speed and diagnostic power. Factors such as reliability and validity are not reported on in this manuscript. 

The validity of the test is of great importance since it is an indicator of how well the test is able to diagnose patients with colour defects as the standard tests do [10]. Although our design located in the preliminary stages, we are planning to evaluate this modified test among adult subjects and its functional performance in children as well. This test should be administered to normal patients, as well as patients with colour vision defects like anomalous trichromats to assess its broader use in future studies. 

Furthermore, the new test’s ability to classify visual colour defects also needs to be assessed. The new test must be able to achieve a high level of sensitivity and reliability to be chosen as a clinical routine screening test in the aforementioned geographical region.

## DISCLOSURE

Conflicts of Interest: None declared.
